# Brain natriuretic peptide suppresses pain induced by BmK I, a sodium channel-specific modulator, in rats

**DOI:** 10.1186/s10194-016-0685-y

**Published:** 2016-09-29

**Authors:** Zheng-Wei Li, Bin Wu, Pin Ye, Zhi-Yong Tan, Yong-Hua Ji

**Affiliations:** 1 Laboratory of Neuropharmacology and Neurotoxicology, Shanghai University, Nanchen Road 333, Shanghai, 200436 People’s Republic of China; 2 Department of Pharmacology and Toxicology and Stark Neurosciences Research Institute, Indiana University School of Medicine, Indianapolis, IN 46202 USA

## Abstract

**Background:**

A previous study found that brain natriuretic peptide (BNP) inhibited inflammatory pain via activating its receptor natriuretic peptide receptor A (NPRA) in nociceptive sensory neurons. A recent study found that functional NPRA is expressed in almost all the trigeminal ganglion (TG) neurons at membrane level suggesting a potentially important role for BNP in migraine pathophysiology.

**Methods:**

An inflammatory pain model was produced by subcutaneous injection of BmK I, a sodium channel-specific modulator from venom of Chinese scorpion Buthus martensi Karsch. Quantitative PCR, Western Blot, and immunohistochemistry were used to detect mRNA and protein expression of BNP and NPRA in dorsal root ganglion (DRG) and dorsal horn of spinal cord. Whole-cell patch clamping experiments were conducted to record large-conductance Ca^2+^-activated K^+^ (BK_Ca_) currents of membrane excitability of DRG neurons. Spontaneous and evoked pain behaviors were examined.

**Results:**

The mRNA and protein expression of BNP and NPRA was up-regulated in DRG and dorsal horn of spinal cord after BmK I injection. The BNP and NPRA was preferentially expressed in small-sized DRG neurons among which BNP was expressed in both CGRP-positive and IB4-positive neurons while NPRA was preferentially expressed in CGRP-positive neurons. BNP increased the open probability of BK_Ca_ channels and suppressed the membrane excitability of small-sized DRG neurons. Intrathecal injection of BNP significantly inhibited BmK-induced pain behaviors including both spontaneous and evoked pain behaviors.

**Conclusions:**

These results suggested that BNP might play an important role as an endogenous pain reliever in BmK I-induced inflammatory pain condition. It is also suggested that BNP might play a similar role in other pathophysiological pain conditions including migraine.

## Background

Chronic pain is a common and serious health problem all around the world. Chronic inflammatory pain resulted from the tissue insult can last for months. The inflammatory pain behaviors induced by venom of Chinese scorpion Buthus martensi Karsch (BmK) include spontaneous pain, ipsilateral thermal hypersensitivity, and bilateral mechanical hypersensitivity in rats [1, 2]. The BmK venom producing inflammatory pain contains various neurotoxins described as specific modulators of voltage-gated sodium channels (VGSCs) [3–5]. Among them, BmK I is a α-like neurotoxin that slows down the inactivation of sodium channels through binding on VGSC receptor site 3 [6–8]. Importantly, BmK I has been demonstrated to be the indispensable contributor for the inflammatory pain caused by the BmK venom.

Natriuretic peptides (NPs) are a family of structurally related peptides derived from several genes, including atrial natriuretic peptide (ANP), brain natriuretic peptide (BNP), and C-type natriuretic peptide (CNP) in mammals. NPs play their roles through binding to specific high affinity receptors on the surface of target cells, including NPRA, NPRB and NPRC [9, 10]. BNP, which was initially identified in porcine brain [11], is considered to act on NPRA, a guanylyl cyclase receptor, and subsequently activates pGC to produce cGMP [12]. cGMP directly opens cyclic nucleotide gated (CNG) channels, and also plays vital physiological roles via activating protein kinase G (PKG) pathway [13].

Accumulating evidences indicate that all NPs and their receptors are widely distributed in a variety of tissues [14–16]. In the central nervous system (CNS), functions of BNP and NPRA range from modulating neuroprotection to synaptic transmission [17]. A previous study found that BNP inhibited the excitability of small dorsal root ganglion (DRG) neurons, pain receptors in the peripheral nervous system, and inflammatory pain induced by CFA and formalin through activating the NPRA/PKG/BK_Ca_ channel pathway [18]. A recent study found that functional NPRA is expressed in almost all the trigeminal ganglion (TG) neurons at the membrane level [19]. Chronic activation of NPRA by BNP down-regulated the excitability of TG neurons.

In the present study, we investigated the role of BNP in the BmK I-induced inflammatory pain. We found that BmK I increased the expression of BNP and NPRA in DRG and spinal cord. The increased expression in DRG was preferentially in small-sized DRG neurons. BNP increased BK_Ca_ currents and suppressed membrane excitability of DRG neurons, and inhibited spontaneous and evoked pain behaviors induced by BmK I.

## Methods

### Animals

Adult male Sprague–Dawley rats were provided by Shanghai Experimental Animal Center, Chinese Academy of Sciences. Rats weighing 220–250 g were used in the behavioral test, immunohistochemistry, Western Blot, and qRT-PCR experiments while rats weighing 80–120 g were used in electrophysiological experiments. Rats were housed in a light/dark cycle of 12 h, at 21–23 °C stable room temperature and 50 % humidity. All animal experiments followed European Community guidelines for the use of experimental animals and the policies issued by the International Association for the Study of Pain [20].

### Inflammatory pain model

The crude BmK venom was purchased from an individual scorpion culture farm in Henan Province, China. BmK I was purified according to previously described procedures [7]. BmK I (dissolved in sterile saline) was intraplantarly (i.pl.) injected into the left side of rat hind paw.

### Preparation and administration of BNP

BNP was purchased from Sigma-Aldrich (St. Louis, MO, USA). BNP was dissolved in saline. As described previously, 10 μl BNP were directly injected by lumbar puncture at the L4-L5 spinal cord [21].

### Behavioral tests

The dose-dependent and time-related effects of BmK I-induced pain-related behaviors were investigated. The rats were randomly divided into 4 groups: (1) rats with i.pl. injection of 10 μg/50 μl BmK I in sterile saline (*n* = 7); (2) rats with i.pl. injection of 10 μg/50 μl BmK I in sterile saline at 0.5 h after intrathecal (i.t.) injection of sterile saline (*n* = 7); (3) rats with i.pl. injection of 10 μg/50 μl BmK I at 0.5, 2, 3, 4 h after i.t. injection of 2 μg/10 μl BNP (*n* = 7); (4) rats with i.pl. injection of 10 μg/50 μl BmK I at 2 h after i.t. injection of 1,2,3 μg/10 μl BNP (*n* = 3). After BmK I injection, pain-related behaviors induced by BmK I were tested at different time points. For the spontaneous pain, rats were continuously observed for 2 h after BmK I injection. And then, the tests for the developmental time window of thermal hypersensitivity were performed at 4, 8 h, and then at 1, 2, 3, 5, 7, and 10 days after injection of BmK I. The tests of the developmental time window of mechanical hypersensitivity were evaluated at 2, 4, 8 h, and then at 1, 2, 3, 5, 7, and 10 days after injection of BmK I. The measurement of rat spontaneous nociceptive responses, paw withdrawal mechanical threshold (PWMT) and paw withdrawal thermal latency (PWTL) of rats described by our previous report [2].

### Real-time quantitative polymerase chain reaction

Total RNA was isolated from ipsilateral and Contralateral L4-L5 spinal cord and DRG of adult male rats (at each time course, *n* = 3) with Total RNA Extractor (Trizol) (Sangon Biotech, Shanghai, China), then reverse-transcribed with Prime-Script®RT Master Mix (TaKaRa, Dalian, China), according to the manufacturer’s protocol. Primer sequences targeted to BNP, NPRA, GAPDH and β-actin were designed by Primer Premier 6.0 software, with sequences respectively were as follows (Table 1). All primers were synthesized by Invitrogen (Shanghai, China). Quantitative PCR was performed in SYBR® Premix Ex TaqTM (TaKaRa, Dalian, China), using CFX96 Touch™ Real-Time PCR Detection System (Bio-Rad). The BNP and NPRA subtypes mRNA was normalized to the average of GAPDH and β-actin mRNA level. Data were analyzed using the delta-delta Ct method.

**Table 1:** Sequences of primers

Primer name	Primer sequences (5′-3′)
*BNP*-*S*	-*AAGGACCAAGGCCCTACAA*-
*BNP*-*A*	-*CGGTCTATCTTCTGCCCAAA*-
*NPRA*-*S*	-*AGGAGATGGGCAGGACAGGA*-
*NPRA*-*A*	-*TCAGGATTATCAGGCTCTTTGT*-
*β*-*actin*-*S*	-*ACTATCGGCAATGAGCGGTTCC*-
*β*-*actin*-*A*	-*AGCACTGTGTTGGCATAGAGGTC*-
*GAPDH*-*S*	-*CAAGTTCAACGGCACAGTCA*-
*GAPDH*-*A*	- *CCATTTGATGTTAGCGGGAT*-

### Western blotting

At different time points (2 h, 8 h, 1 day, 2 days, 5 days, 7 days) after i.pl. BmK I injection, the rats were anesthetized by intraperitoneal injection of pentobarbital sodium (60 mg/kg). The L4-L5 spinal cord and DRG were rapidly removed, and stored at −80 °C. Their protein lapping liquids were obtained by homogenization in ice-cold RIPA Lysis Buffer (Beyotime, Shanghai, China). The supernatant was collected after centrifugation at 12000 r/min for 15 min. Protein concentration was measured using BCA Protein Assay Kit (Biotech Well, Shanghai, China). SDS-PAGE Sample Loading Buffer was mixed into the supernatant by proportion and the mixture was bathed for 5 min at boiling water. Protein samples (20 μl) were concentrated on 6 % SDS-PAGE concentrated gels and separated on 10 % (for macromolecular protein) or 15 % (for small molecular protein) SDS-PAGE separation gels and blotted on a polyvinylidene fluoride membrane (Merck Millipore, Germany). The membranes were blocked in nonfat milk solution for 2 h, and then incubated overnight at 4 °C with primary antibodies solution: goat polyclonal anti-BNP (1:200; Santa Cruz, CA, USA), rabbit polyclonal anti-NPRA (1:300, Abcam, MA, USA), mouse polyclonal anti-β-actin (1:500; Santa Cruz, CA). Next day, the membranes were incubated with primary antibodies solution for 2 h at room temperature (RT) and washed using PBST (5-10 min) for three times, then incubated for 1.5 h at RT with secondary antibodies solution: goat anti-rabbit IgG(H + L) HRP(1:12 000; Kangchen, CA), rabbit anti-goat IgG(H + L) HRP(1:10 000; Kangchen, CA), goat anti-mouse IgG(H + L) HRP(1:10 000; Kangchen, CA). The blots were ignited in ECL reagent (Merck Millipore, Germany), and then developed in fully automatic chemiluminescence image analysis system (Tanon, Shanghai, China). Gel image system 1D Analysis Software(Tanon) was used for their densitometric quantification. The standardization ratio of BNP or NPRA to β-actin band densitometric data was used to calculate the change of the expression.

### Immunohistochemistry

Two days after i.pl. BmK I injection, the adult rats were anesthetized by pentobarbital sodium, then perfused with sterile saline and 4 % paraformaldehyde in 0.1 mol/L phosphate buffer (PB). The rat L4-L5 lumbar spinal cord and DRG tissues was post-fixed and cryoprotected in 20 % sucrose 0.1 mol/L PB solution, then moved into the 30 % sucrose solution. Tissue transverse frozen sections (20 μm thick) were cut on CM1900 freezing microtome (Leica, Germany) and stored at −20 °C. Frozen sections were in the wet air for 0.5 h and were incubated with 5 % goat serum (in PBS) for 2 h at RT followed by incubation overnight at 4 °C with primary antibodies diluent (contain 0.3 % Triton X-100): goat polyclonal anti-BNP (1:50; Santa Cruz, CA, USA), rabbit polyclonal anti-NPRA (1:100; Abcam, MA, USA), mouse monoclonal anti-NeuN (1:500; Millipore Bioscience Research Reagents, DA, GER), Mouse monoclonal to calcitionin gene related peptide (CGRP) (1:100; Abcam, MA, USA), fluorescein-conjugated isolectin-B4(IB4) (1:400; Sigma), Mouse monoclonal Anti-Neurofilament 200 (NF200) (1:800; Sigma). Before incubation with secondary antibodies, the sections were washed using PBS (5-10 min) for three times. After 2 h of incubation with secondary antibodies: goat anti-rabbit IgG, Cy3 conjugate (1:400; Merck Millipore), rabbit anti-goat IgG, Cy3 conjugate (1:100; Biotech Well), donkey anti-mouse IgG Fluorescein (FITC) conjugate (1:200; Jackson Immunoresearch), the sections were washed using PBS (5-10 min) for three times. Digital images were captured from laser scanning confocal microscope (Leica, Germany) and then merged by Image J software.

### Electrophysiology

The rats were anesthetized with ether and decapitated. DRGs were dissected from the L4-L5 lumbar region. Neurons were acutely isolated from DRGs with 1 mg/ml collagenase type 1A, 0.6 mg/ml trypsin type I, in D-Hanks at 37 °C for 30 min. Single cells were dissociated mechanically with a series of fire-polished Pasteur pipettes, and plated on glass slides covered with Poly-D-Lysine, then placed into dishes. The cells were cultured for 2 h in Dulbecco’s modified Eagle medium (DMEM F12; Gibco, Invitrogen, Grand Island, NY, USA). Culture dishes were incubated at 37 °C in a humidified atmosphere containing 5 % CO_2_. Patch clamp was performed within 0-10 h after culturing. All of the recordings were made from small-diameter (<25 μm) DRG neurons. External solution (ECS) contained (mM): 5 KCl, 150 NaCl, 2 CaCl_2_, 1.5 MgCl_2_, 10 HEPES, and 10 glucose, pH 7.4. 2, 1.75, 1.5 and 2.0 mM CaCl_2_ was replaced by the same concentration of MgCl_2_ to prepare ECS containing 0, 0.25, 0.5 and 1 mM Ca^2+^. The pipette solution contained (mM): 140 KCl, 1 MgCl_2_, 2 CaCl_2_, 5 EGTA, 10 HEPES, 2 Na-ATP, and 0.3 Na-GTP, pH 7.3. ECS were saturated with O_2_ before using. BK_Ca_ channel blocker: iberiotoxin (IBTX). Whole-cell path clamping experiments were performed using an EPC-10 amplifier (HEKA eletronik, Germany) at room temperature. Patch pipettes were fabricated from glass capillary tubes by PP-830 Puller (Narishige, Japan) with the resistance of 3–5 MΩ. Data acquisition and stimulation protocols were controlled by Pulse/PusleFit 10.0 software (HEKA Elektronik). Before “breakthrough” with additional suction, seal resistance was more than 1GΩ. After whole-cell mode was established, input resistance was larger than 200 MΩ.

### Statistical analysis

The data were analyzed with GraphPad prism 5.0 Software and Origin 8.5 Software. All data were presented as means ± SEM (standard error). Statistical analysis of the data was performed using one-way ANOVA followed by Fisher’s PLSD test, two-way ANOVA followed by Boferroni or unpaired Student’s t-test. Values were considered as statistically significant at *P* < 0.05.

## Results

### Up-regulation of BNP and NPRA in DRG and spinal cord in the BmK I-induced pain models

**Fig. 1:**
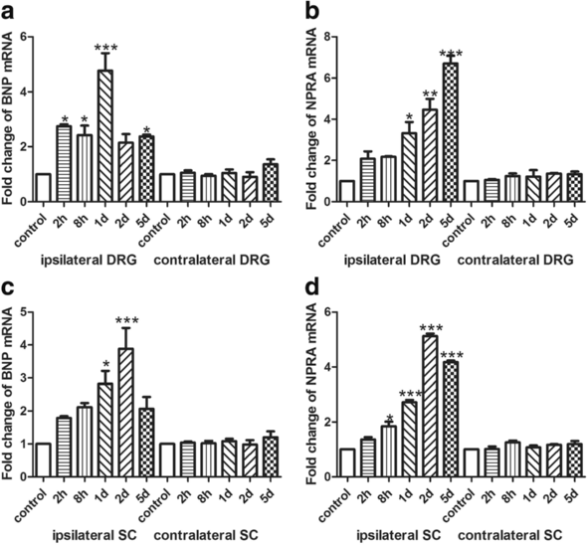
The mRNA expression of BNP and NPRA in DRG and spinal cord at different time points after BmK I injection. **a**: The mRNA expression level of BNP in bilateral sides of L4-L5 DRG. **b**: The mRNA expression level of NPRA in bilateral sides of L4-L5 DRG. **c**: The mRNA expression level of BNP in bilateral sides of L4-L5 spinal cord. **d**: The mRNA expression level of NPRA in bilateral sides of L4-L5 spinal cord. *P < 0.05, **P < 0.01, ***P < 0.001 compared with control groups (*n* = 3). Error bars indicated S.E.M

The mRNA expression of BNP and NPRA before and after BmK I injection was firstly examined in DRG and spinal cord using real-time, quantitative PCR technique. As shown in Fig. 1, BmK I caused significant increase in mRNA expression of BNP (Fig. 1a & c) and NPRA (Fig. 1b & d) at multiple time points after injection in ipsilateral DRG and spinal cord. The mRNA expression of BNP and NPRA was not significantly changed at any time points after BmK I injection in either contralateral DRG or spinal cord. The time course of BmK I increasing mRNA expression of BNP and NPRA appeared different in DRG (Fig. 1a & b). The expression of BNP was increased by about 2.5 times at 2 h and 8 h after BmK I injection compared to control. At day1, the expression of BNP peaked at a level about 5 times higher than control. At day2 and day5, the peaked expression reduced to a level similar to 2 h and 8 h. In contrast to this transient-peak pattern of BNP, the expression of NPRA increased gradually after BmK I injection and reached peak at day5. Compared to DRG, the time course of BmK I-increased expression of BNP and NPRA was similar in spinal cord (Fig. 1c & d). Both BNP and NPRA showed a peak expression at day2 after BmK I injection.

**Fig. 2:**
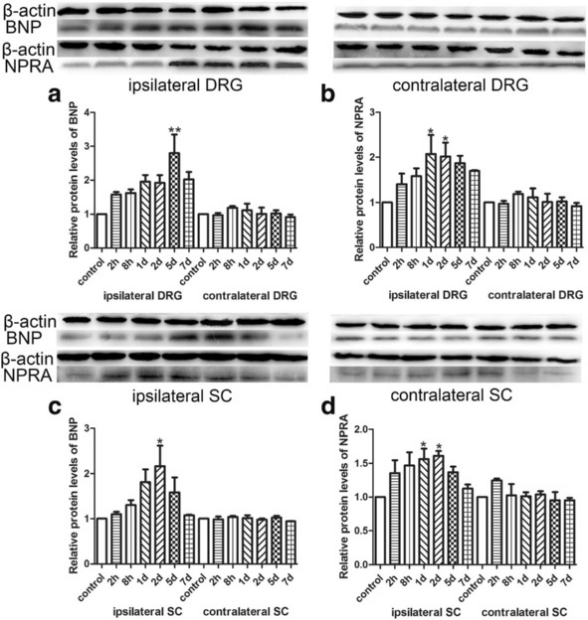
Western blot analysis showed the protein expression of BNP and NPRA in DRG and spinal cord at different time points after BmK I injection. **a**: The protein expression of BNP in bilateral sides of L4-L5 DRG. **b**: The protein expression of NPRA in bilateral sides of L4-L5 DRG. **c**: The protein expression of BNP in bilateral sides of L4-L5 spinal cord. **d**: The protein expression of NPRA in bilateral sides of L4-L5 spinal cord. *P < 0.05, **P < 0.01, ***P < 0.001 compared with control groups (*n* = 3). Error bars indicated S.E.M

The effects of BmK I on the expression of BNP and NPRA was further studies at protein level using Western Blotting technique. As shown in Fig. 2, BmK I significantly increased protein expression of BNP (Fig. 2a & c) and NPRA (Fig. 2b & d) at some time points after injection in ipsilateral DRG and spinal cord. The protein expression of BNP and NPRA was not significantly changed at any time points after BmK I injection in either contralateral DRG or spinal cord. The time course of BmK I increasing protein expression of BNP and NPRA appeared different in DRG (Fig. 2a & b). The protein expression of BNP was peaked at day5 while the expression of NPRA was peaked at day1. In spinal cord, the BmK I-caused increase in protein expression was peaked at day2 after injection for both BNP and NPRA (Fig. 2c & d).

**Fig. 3:**
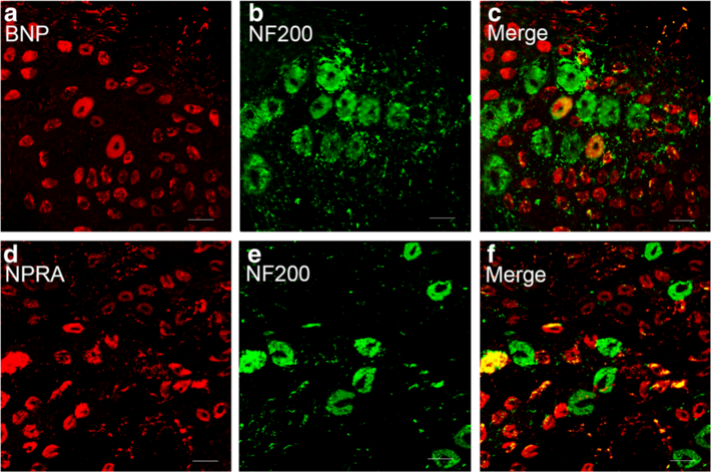
Representative microphotographs showed the location and expression of BNP and NPRA in NF200-positive DRG neurons. Almost all immunofluorescence staining for BNP and NPRA (**a**, **d**) was not co-localized (**c**, **f**) with NF200 (**b**, **e**) (*n* = 3). Scale bar, 50 μm

**Fig. 4:**
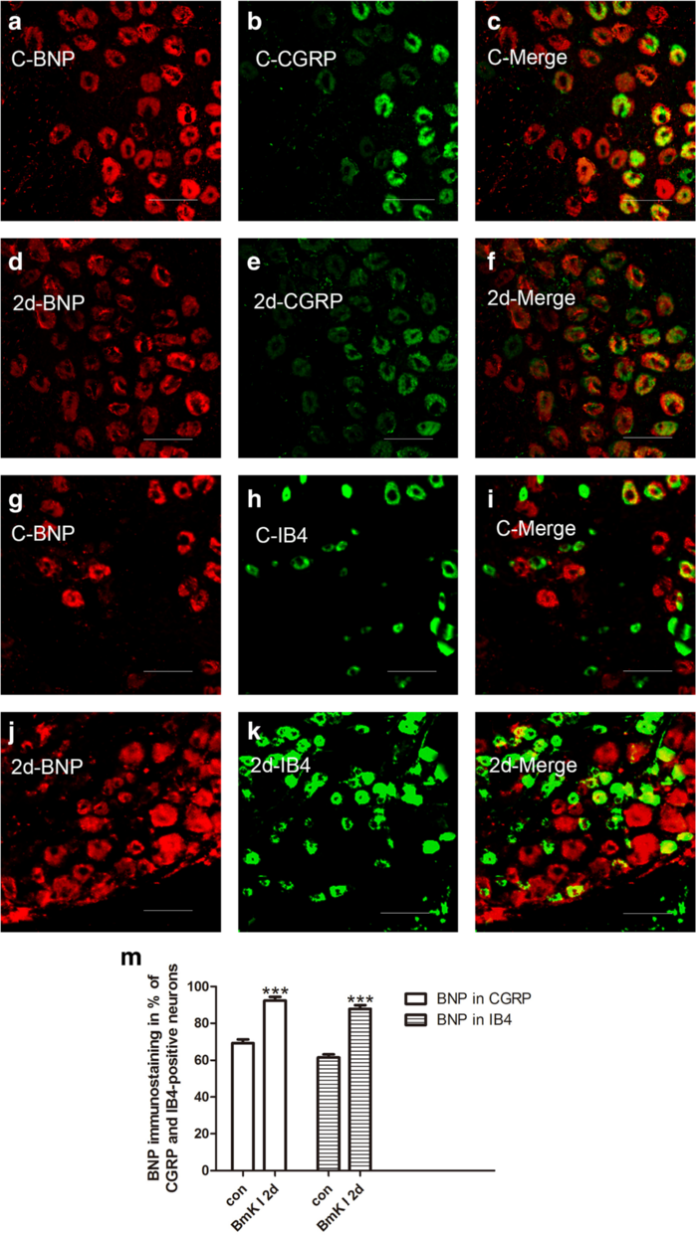
Representative microphotographs showed the location and expression of BNP in CGRP-positive and IB4-positive small DRG neurons. Immunofluorescene staining for BNP (**a**, **d**) and CGRP (**b**, **e**) were co-localized (**c**, **f**) in control (**a**-**c**) at 2 days after i.pl. BmK I injection (**d**-**f**). Immunofluorescene staining for BNP (**g**, **j**) and IB4 (**h**, **k**) were co-localized (**i**, **l**) in control (**g**-**i**) at 2 days after i.pl. BmK I injection (**j**-**i**). **m**, ratio of CGRP-positive and IB4-positive small DRG neurons co-localized with BNP between control group and the group of 2 days after i.pl. BmK I injection. Scale bar, 50 μm

**Fig. 5:**
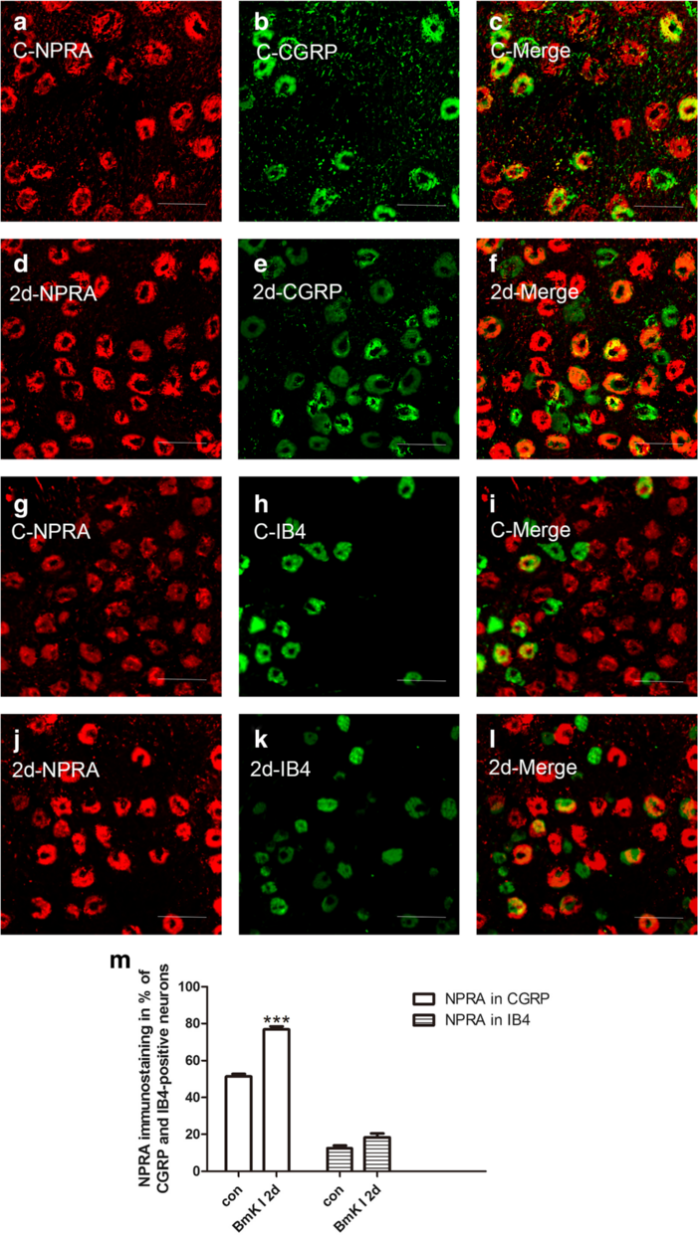
Representative microphotographs showed the location and expression of NPRA in CGRP-positive and IB4-positive small DRG neurons. Immunofluorescene staining for NPRA (**a**, **d**) and CGRP (**b**, **e**) were co-localized (**c**, **f**) in control (**a**-**c**) at 2 days after i.pl. BmK I injection (**d**-**f**). Immunofluorescene staining for NPRA (**g**, **j**) and IB4 (**h**, **k**) were co-localized (**i**, **l**) in control (**g**-**i**) at 2 days after i.pl. BmK I injection (**j**-**i**). **m**, Ratio of CGRP-positive and IB4-positive small DRG neurons co-localized with NPRA between control group and the group of 2 days after i.pl. BmK I injection. Scale bar, 50 μm

To study the BmK I-increased expression of BNP and NPRA in different neuronal populations, immunohistochemical experiments were conducted on tissue slices of DRG. Double staining of NF200 (marker for myelinated, large fiber/soma in DRG) and BNP or NPRA was first examined. As showed in Fig. 3, both BNP and NPRA were preferentially expressed in the small DRG neurons while NF200 was preferentially expressed in the large DRG neurons. To further study neuronal subpopulations in small-sized DRG neurons, staining markers CGRP and IB4 were used to label the peptidergic neurons and non-peptidergic neurons, respectively. It was found that BNP was expressed in both CGRP-positive and IB4-positive neurons while NPRA was preferentially expressed in CGRP-positive neurons (Fig. 4a-c and g-i, Fig. 5a-c and g-i). Two days after BmK I injection, the immunostaining of BNP was significantly increased in both CGRP-positive and IB4-positive neuronal subpopulations (Fig. 4m). On the other hand, the immunostaining of NPRA was selectively increased in the CGRP-positive neurons compared to IB4-positive neurons (Fig. 5m).

**Fig. 6:**
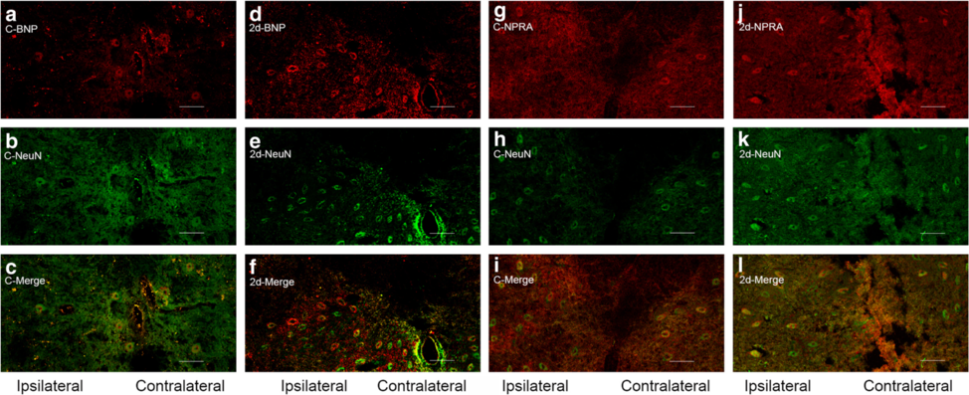
Representative microphotographs showed the location and expression of BNP and NPRA in NeuN-positive spinal cord neurons. Immunofluorescene staining for BNP (**a**, **d**) and NeuN (**b**, **e**) were co-localized (**c**, **f**) in control (**a**-**c**) at 2 days after i.pl. BmK I injection (**d**-**f**). Immunofluorescene staining for NPRA (**g**, **j**) and NeuN (**h**, **k**) were co-localized (**i**, **l**) in control (**g**-**i**) at 2 days after i.pl. BmK I injection (**j**-**i**). Scale bar, 100 μm

As shown in Fig. 6, BNP and NPRA were double-immunofluorescent with a neuron marker (NeuN) in spinal cord. It was found that the immunofluorescence staining of both BNP and NPRA was increased in ipsilateral but not in contralateral spinal cord at 2 days after i.pl. BmK I injection.

### Elevating the open probability of BK_Ca_ channels and suppressing excitability of small DRG neurons by BNP

**Fig. 7:**
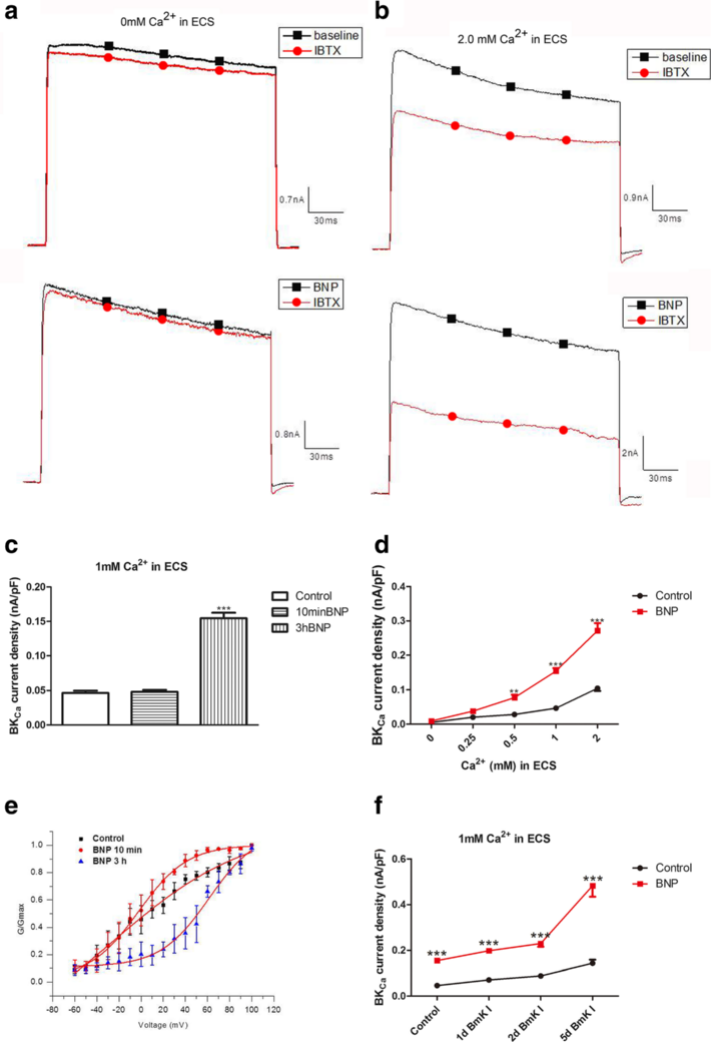
BNP increased the open probability of BK_Ca_ channels. **a**: An outward current in small DRG neurons were induced by a voltage ramp from −60 to 100 mV (0.2 s duration) in whole-cell voltage-clamp configuration. In Ca^2+^-free ECS BNP did not affect the current, IbTX did not change the current significantly either. **b**: In small DRG neurons incubated in ECS with 1 mM Ca^2+^, the current was elevated by pretreatment of BNP (100 ng/ml) for more than 2 h and was inhibited by BK_Ca_ channel inhibitor IbTX (100 nM). **c**: In small DRG neurons incubated in ECS with 1 mM Ca^2+^, BK_Ca_ current was elevated by pretreated with BNP (100 ng/ml) for 3 h (*n* = 7), but not by bath-applied BNP (100 ng/ml) for 10 min (*n* = 6). **d**: BK_Ca_ current was induced in small DRG neurons incubated in ECS with 0.5, 1 or 2 mM Ca^2+^, and this current was increased by pretreatment of BNP for 3 h (*p* < 0.01, *n* = 7 for 0.5 mM Ca^2+^ at 90 mV; *p* < 0.001, *n* = 7 for 1, 2 mM Ca^2+^ at 90 mV). In the presence of 0 or 0.25 mM extracellular Ca^2+^, BK_Ca_ current was not induced and BNP did not have effect (*n* = 7 neurons/group). BK_Ca_ current was calculated as the difference between total current and IBTX-resistant current, and was normalized to the cell membrane capacitance. **e**: G(V) relationship indicated that treatment of BNP for 10 min did not enhance the activation of BK_Ca_ currents which was significantly rightward shifted after treatment of BNP for 3 h. **f**: 1 day, 2 days and 5 days after i.pl. BmK I injection, BK_Ca_ current density of small DRG neurons were elevated significantly compared with control rats. **p < 0.01 and ***p < 0.001 versus control. Error bars indicated SEM

A voltage stimulation depolarizing from −50 to 90 mV (0.2 s duration) was used to trigger outward currents in small DRG neurons. A selective BK_Ca_ channel blocker IBTX (100 nM) was used to block BK_Ca_ currents (Fig. 7a & b). The outward currents blocked by 100 nM IBTX were defined as BK_Ca_ currents. To test the effects of BNP on the BK_Ca_ currents, BNP (100 ng/ml) were pretreated for 3 h in culture medium. Acute application of BNP at the same concentration for 10 min did not change BK_Ca_ currents (Fig. 7c). At 0 extracellular Ca^2+^ condition, the BK_Ca_ currents were not activated in the either absence or presence of BNP (100 ng/ml) (Fig. 7a & d). Increasing extracellular Ca^2+^ from 0.25 to 2 mM gradually increased the density of the BK_Ca_ currents in the either absence or presence of BNP (100 ng/ml) (Fig. 7b & d). Compared to control, 100 ng/ml BNP significantly increased the density of BK_Ca_ currents at 0.5, 1, and 2 mM extracellular Ca^2+^ conditions (Fig. 7d, *n* = 7). The activation curve of the BK_Ca_ currents was shifted by BNP positively. The midpoint of activation (V_1/2_) was significantly rightward shifted from −2.38 ± 1.60 to 61.39 ± 4.95 and the slope factor (km) was increased from 15.12 ± 1.47 to 23.02 ± 3.27 (Fig. 7e). The increasing effects of BNP on BK_Ca_ current density of small DRG neurons were similar at 1 or 2 days after BmK I injection compared to pre-injection control (Fig. 7f, *n* = 5). However, BNP caused a larger increase in BK_Ca_ current density at 5 days after BmK I injection compared to pre-injection, 1 day and 2 days after BmK I injection (Fig. 7f).

**Fig. 8:**
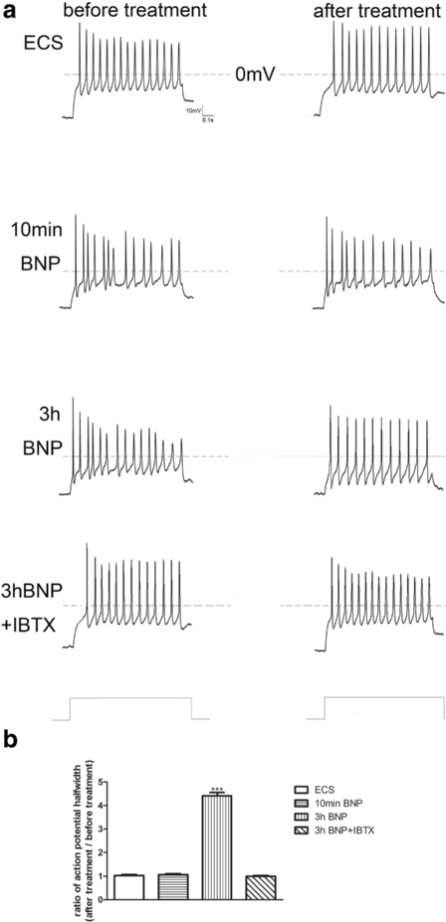
BNP reduced the excitability of small DRG neurons. **a**: The representative action potentials triggered by depolarizing current stimulation (1 s duration; 200 pA) in the absence (before treatment) and presence (after treatment) of treatments including extracellular solution condition (ECS), 10 min BNP, and 3 h pretreatment of BNP without or with IBTX, respectively. **b**: The ratio of action potential halfwidth in small DRG neurons in the presence (after treatment) vs. absence (before treatment) of treatments. The action potential halfwidth was measured from the first action potential triggered by current injection. ***p < 0.001, treatment versus control. Error bars indicated S.E.M

Current-clamp recordings were employed to determine the effect of BNP on the excitability of DRG neurons. A depolarizing current (1 s duration, 200 pA) was injected into small DRG neurons to trigger action potentials. The effects of 10 min ECS, 10 min BNP, pretreatment of BNP for 3 h, and pretreatment of BNP for 3 h + IBTX were examined in the absence (“before treatment”, Fig. 8a-left) and presence of treatments (“after treatment”, Fig. 8a-right). Application of BNP significantly lowered the number of action potentials (from 15.4 ± 1.2 to 12.3 ± 1.4; *n* = 12). The decreasing effect of BNP on action potential number was prevented by pretreatment of 100 n M IBTX (Fig. 8a). The ratio of the action potential halfwidth (after treatment vs before treatment) was not altered by application of BNP for 10 min (1.060 ± 0.042; *n* = 12). However, pretreatment of BNP for 3 h significantly increased the ratio to 2.002 ± 0.078 (*n* = 12) compared to ECS (1.031 ± 0.042; *n* = 12). The increasing effect of BNP on ratio halfwidth was prevented by pretreatment of 100 n M IBTX (0.992 ± 0.034; *n* = 12) (Fig. 8b).

### Inhibition of BNP on BmK I-induced inflammatory pain related behaviors

The effects of intrathecal injection of BNP on BmK I-induced pain behaviors were studied. Both spontaneous pain behaviors and evoked pain behaviors were studied. The spontaneous pain behaviors were studied in the first two hours after BmK I injection while the evoked pain behaviors were studied from 4 h to 10 days after BmK I injection. The evoked pain behaviors were not studied within the 4 h after BmK I injection to avoid the overlapping with the spontaneous pain behaviors.

**Fig. 9:**
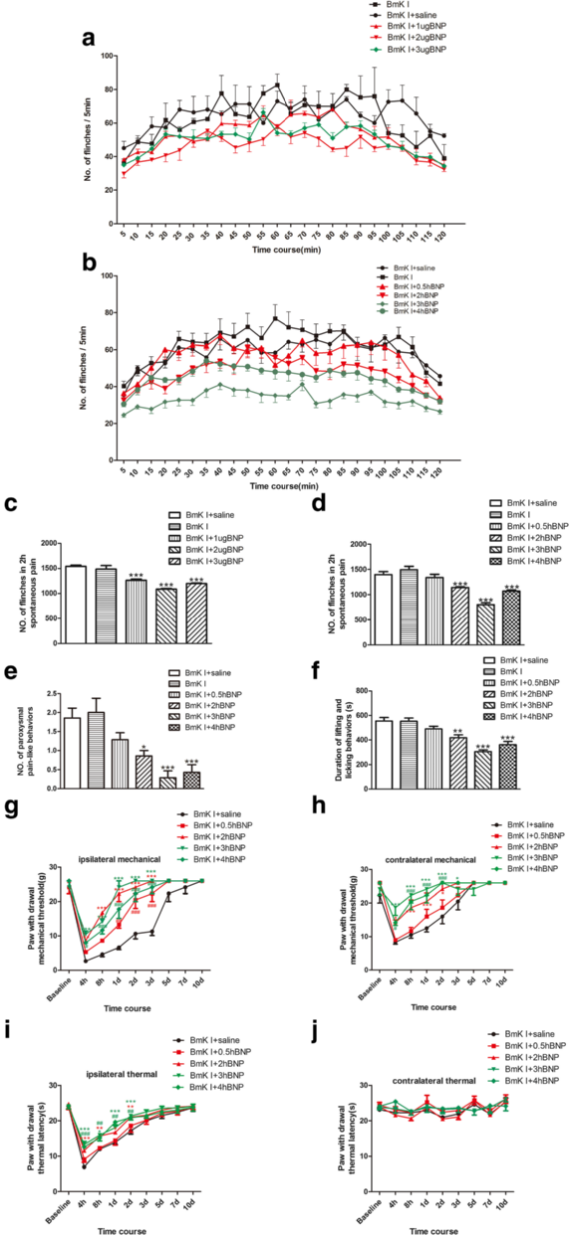
BNP (i.t.) suppressed BmK I-induced inflammatory pain-related behaviors. **a**: Rat flinch behavior was attenuated by pretreatment of 10 μl saline or BNP(1, 2 or 3 μg in saline) at 2 h before BmK I administration. **b**: Rat flinch behavior was attenuated by pretreatment of 10 μl saline or BNP(2 μg in saline) at 0.5, 2, 3, 4 h before BmK I administration. **c**: Suppression of total number of the rat paw flinches by 10 μl saline or BNP(1, 2 or 3 μg in saline) during 2 h after BmK I injection. **d**: Suppression of total number of the rat paw flinches by 10 μl saline or BNP(2 μg in saline) during 2 h after BmK I injection. **e**: Suppression of total number of paroxysmal pain-like behaviors by 10 μl saline or BNP(2 μg in saline) during 2 h after i.pl. BmK I injection. **f**: Suppression of duration of lifting and licking behaviorsby 10 μl saline or BNP(2 μg in saline) during 2 h after i.pl. BmK I injection. Ipsilateral mechanical hyperalgesia (**g**), contralateral mechanical hyperalgesia (**h**) and ipsilateral thermal hyperalgesia (**i**) were suppressed by 10 μl saline or BNP(2 μg in saline) pretreatment for 0.5, 2, 3, 4 h. **j**: There was no difference of conltralateral basal thermal latency among five groups. Rat hindpaw injected with BmK I was considered as ipsilateral side, and the other side was named as contralateral side. All data were showed as mean ± S.E.M. (**a**, **c**: n = 3; **b**, **d**-**j**: *n* = 7). *P < 0.05, **P < 0.01, ***P < 0.001, #*P* < 0.05, ##*P* < 0.01, ###*P* < 0.001, compared with BmK I + saline group

Intrathecal injection of BNP significantly inhibited the spontaneous pain behaviors induced by subcutaneous injection of BmK I. As shown in Fig. 9a and Fig. 9c, intrathecal injection of BNP 2 h before intraplantar BmK I injection significantly inhibited flinching from control value of 1543 ± 21 to 1267 ± 21.9 (*p* < 0.001), 1088 ± 14.5 (p < 0.001) and 1198 ± 12.3 (*p* < 0.001) for 1ug, 2ug and 3ug of BNP, respectively; *n* = 3 rats/group). To study time course of intrathecal BNP on the spontaneous pain behaviors produced by BmK I, BNP were injected intrathecally at 0.5 h, 2 h, 3 h, 4 h before subcutaneous injection of BmK I. Spontaneous pain behaviors including flinching, paroxysmal, lifting and licking of rats were tested. As shown in Fig. 9b, d-f, BNP significantly inhibited the number of flinching and paroxysmal behaviors, and the duration of lifting and licking at 2–4 h before BmK I injection. The peak inhibition occurred at 3 h before BmK I injection for the spontaneous pain behaviors.

In addition to the acute spontaneous pain behaviors, intrathecal injection of BNP significantly inhibited the evoked pain behaviors over the time course from 4 h to 10 days after BmK I injection. As shown in Fig. 9g-i, intrathecal BNP significantly increased the threshold and shortened the time course of paw withdrawal for ipsilateral and contralateral mechanical stimulation, and for ipsilateral thermal stimulation. Paw withdrawal threshold for contralateral thermal stimulation was not significantly changed by BmK I injection in the absence or presence of intrathecal BNP (Fig. 9j).

## Discussion

**Fig. 10:**
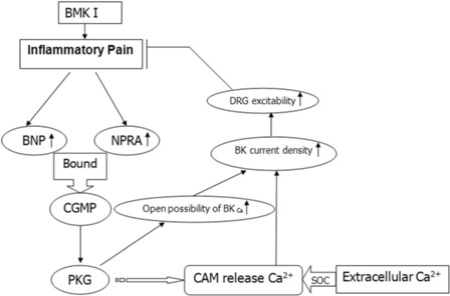
Flow chart of BNP signaling pathway induced by BmK I

This study showed that BNP and NPRA were expressed in small DRG and spinal cord neurons, and were upregulated after i.pl. BmK I injection. BNP was expressed in both CGRP-positive and IB4-positive neurons while NPRA was preferentially expressed in CGRP-positive neurons in DRG. In vitro patch clamping experiments found that BNP suppressed the membrane excitability through increasing the open probability of the BK_ca_ currents. Furthermore, intrathecal injection of BNP significantly inhibited BmK I-induced nociceptive responses (Fig. 10). Therefore, BNP secreted from nociceptive afferent neurons might be an endogenous analgesic molecule for BmK I-induced inflammatory pain. Considering the functional expression of BNP/NPRA signal system in both DRG and TG neurons, the activation of BNP signaling pathway might have a broad prospect in recovering chronic pain conditions including somatic pain and migraine [22–24].

### The cGMP/PKG signaling pathway was involved in inhibition of BNP on inflammatory pain

Most of neuropeptide-induced presynaptic inhibition were mediated by G-protein-coupled receptors [25], whereas NPRA was a guanylyl cyclase and did not bind to G-protein. This might represent a novel mechanism for regulating nociceptive afferent transmission. G-protein-coupled receptor mediated signaling pathway was thought to be a rapid response. However, NPRA and receptor tyrosine kinase (RTK) were both enzyme-linked receptors, which can attribute to a slow response. There were many converted steps in signaling via RTK, and some of them were much slower [26]. Similar to this, signaling via NPRA might be very slow. The results of our behavior test confirmed that the inhibition of BNP in the inflammation pain was slow.

Activation of NPRA resulted in addition of cGMP/PKG signaling [18]. The NPRA-mediated cGMP could be elevated by different phosphodiesterase inhibitors [27]. Coordinately, inhibition of cGMP degradation reduces inflammatory pain induced by formalin after intrathecal injection of the phosphodiesterase five inhibitor sildenafil [28, 29]. Thus, the nociceptive afferent transmission might be inhibited by BNP that raised intracellular concentration of cGMP.

Just as the expression of NPRA, the PKG type I (cGKI) was expressed in small DRG neurons and their afferent neurons in the dorsal spinal cord [30]. Cysteine-rich protein 2 (CRP2), a downstream effector of the cGKI, was also expressed in cGKI-containing afferent neurons in the spinal dorsal horn. Nociceptive response of CRP2-knockout mice was elevated in inflammatory pain model, which indicated that CRP2 contributed to inhibition in pain transmission [31]. Therefore, the PKG signaling pathway might negatively regulating nociceptive afferent transmission.

### DRG excitability was reduced by BNP via enhancing the current of BK_Ca_ channels

The open probability of BK_Ca_ channel was enhanced after activating NPRA/cGMP/PKG signaling pathway, and the cell membrane was hyperpolarized through phosphorylation of BK_Ca_ channel [32–34]. Specifically, cGMP activated the activity of PKG, and then phosphorylated BK_Ca_ channel at Ser1134 to regulate BK_Ca_ channel activity [35]. The suppression induced by BNP was blocked after treated with IBTX, a BK_Ca_ channel blocker, indicated that BK_Ca_ channel was downstream of activating NPRA/cGMP/PKG signaling pathway. Activation of NPRA increased intracellular cGMP level by stimulating particulate GC, leading to calcium release from intracellular store through a ryanodine-sensitive pathway [36]. After calcium releasing from intracellular store, calcium was compensated via store-operated channels (SOC) [37, 38]. Activation of SOC caused calcium influx and additional increase in intracellular calcium, resulting in an increase in BK_Ca_ current density and reduction in DRG excitability.

### Stimulation of BNP negatively regulated inflammation pain

The current studies found that the mRNA and protein expression of BNP and NPRA were elevated in small DRG and spinal cord neurons following BmK I injection. It was previously found that subcutaneous injection of BmK I caused release of various biologically active signaling molecules including proinflammation cytokines and brain-derived neurotrophic factor [39–41]. These findings suggest that some of the molecules stimulated by BmK I might activate and cause secretion of BNP from nociceptive afferent neurons resulting in an inhibitory effect on the inflammatory pain.

Three doses of BNP were used to examine the effects of BNP on spontaneous flinching (Fig. 9a & c). Although the inhibitory effect of BNP was increased from 1 ug to 2 ug, but was almost unchanged when the dose was further increased to 3 ug. We suspected that it could be either the effects of BNP was saturated at 2 ug or a reversing effect developed at 3 ug of BNP. Considering the 3 ug BNP caused a smaller inhibition compared to 2 ug BNP (Fig. 9c), the latter possibility could be more likely to be true.

Previously we have reported that BmK I induced a mirror-image mechanical hypersensitivity where injection of BmK I at one side of hind paws caused mechanical hypersensitivity on both sides of hind paws. In the current study, we found that BmK I selectively up-regulated the expresion of BNP and NPRA at the ipsilateral side but not at the contralateral side. Similarly, intrathecal BNP selectively suppressed the spontaneous and evoked pain behaviors at the ipsilateral side of rats. These results suggested that BNP might not involve in the contralateral side of the mirror-image mechanical hypersensitivity induced by BmK I.

### BNP played a role in the relevant relation between pain and itch

The present results showed that the increased expression of BNP and NPRA were increased and that the increased expression might inhibit the pain response after peripheral inflammation. It has been reported that BNP was involved in the itch transmission in spinal cord. BNP can activate spinal NPRA-expressing neurons which then release gastrin releasing peptide (GRP). The released GRP subsequently activate GRP receptor-expressing neurons to relay itch information from the periphery to the brain [42]. It is well know that when pain is relieved during tissue healing, itch appears. The results in the current study suggest that the increased BNP and NPRA might be involve in both pain-relieving and itch following tissue insult.

## Conclusion

BNP and its receptor NPRA were expressed in small DRG neurons and spinal cords neurons of rats. BNP and NPRA were elevated after peripheral tissue inflammation induced by BmK I. Furthermore, intrathecal application of BNP suppressed the excitability of small DRG neurons and alleviated inflammatory pain by activation of BNP signaling pathway. Based on these results, we conclude that BNP and NPRA might serve as endogenous pain-relieving signal system in BmK I-induced inflammatory pain. Future studies will determine whether BNP/NPRA system plays a role in other pathophysiological pain conditions including migraine.
